# NeEMO: a method using residue interaction networks to improve prediction of protein stability upon mutation

**DOI:** 10.1186/1471-2164-15-S4-S7

**Published:** 2014-05-20

**Authors:** Manuel Giollo, Alberto JM Martin, Ian Walsh, Carlo Ferrari, Silvio CE Tosatto

**Affiliations:** 1 Department of Biomedical Sciences, University of Padova, Viale G. Colombo 3, 35131 Padova, Italy; 2 Department of Information Engineering, University of Padova, Via Gradenigo 6, 35121 Padova, Italy

## Abstract

**Background:**

The rapid growth of un-annotated missense variants poses challenges requiring novel strategies for their interpretation. From the thermodynamic point of view, amino acid changes can lead to a change in the internal energy of a protein and induce structural rearrangements. This is of great relevance for the study of diseases and protein design, justifying the development of prediction methods for variant-induced stability changes.

**Results:**

Here we propose NeEMO, a tool for the evaluation of stability changes using an effective representation of proteins based on residue interaction networks (RINs). RINs are used to extract useful features describing interactions of the mutant amino acid with its structural environment. Benchmarking shows NeEMO to be very effective, allowing reliable predictions in different parts of the protein such as β-strands and buried residues. Validation on a previously published independent dataset shows that NeEMO has a Pearson correlation coefficient of 0.77 and a standard error of 1 Kcal/mol, outperforming nine recent methods. The NeEMO web server can be freely accessed from URL: http://protein.bio.unipd.it/neemo/.

**Conclusions:**

NeEMO offers an innovative and reliable tool for the annotation of amino acid changes. A key contribution are RINs, which can be used for modeling proteins and their interactions effectively. Interestingly, the approach is very general, and can motivate the development of a new family of RIN-based protein structure analyzers. NeEMO may suggest innovative strategies for bioinformatics tools beyond protein stability prediction.

## Introduction

The development of Next Generation Sequencing technologies has a tremendous impact on the discovery of missense variants. In humans, dbSNP [1] reports more than one million such variants, while only 1% of them have functional annotation or are referenced in the literature. This gap represents a problem for understanding disease development [2], as the proper characterization of variant effects may require expensive experiments. This is not only important for healthcare, but also for biotechnology, where alanine-scanning mutagenesis is commonly used to study the effect of amino acid variants on protein function and interactions [3]. Finally, designing mutants for protein design [4] and to evaluate their effects on function requires a deeper understanding of the mechanisms by which single variants affect stability. The Gibbs free energy (ΔG) defines the thermodynamic energy of folding compared to the denatured state. The difference between wild type and mutant polypeptide energy (ΔΔG = ΔGwt - ΔGmut) is a measure of how the amino acid change affects protein stability. Polypeptide chains are held together by non-covalent interactions between the residues forming them. The most relevant factors affecting protein folding and stability are hydrogen bonds, van der Waals, electrostatic and hydrophobic interactions, backbone angle preferences and protein chain entropy [5]. Interestingly, the assessment of stability changes has been shown to be critical for the interpretation of variants in key proteins like *TP53 *[6], which is known to have a strong connection with cancer development. In order to help understand the impact of amino acid changes, the ProTherm database [7] collects the free Gibbs energy for thousands wild type and mutant proteins. This source of information is critical for the development of new methods that try to fill the gap of unannotated variants. For the last 15 years, a number of computational tools have been developed for the prediction of stability changes in mutant proteins. Energy-based methods are based on two main approaches [8]. The first type is based on the use of molecular (or quantum mechanic) force fields that try to reflect the physical energy of molecules [9,10]. The second type, also known as knowledge-based potential functions (KBPFs), are energy functions based on statistics computed on sets of experimental or artificially generated protein structures. Most KBPFs rely on a weighted combination of several statistical terms, as in Eris [11] or FoldX [12]. In particular, the latter considers nine different terms like van-der-Waals contributions, solvation energy, hydrogen bonds and the entropy cost. All terms are linearly combined after fitting to experimental data [12].

A completely different approach is adopted by machine learning algorithms (ML). Rather than trying to explicitly describe complex models of thermodynamic energy, they are trained by minimizing the classification error on a reference dataset. A number of ML tools have been proposed for stability prediction of variants, like AutoMute [13], I-Mutant [14,15], MuPro [16] and PoPMuSiC 2.0 [17]. Most of these simulate the change by replacing the side chain of the mutated residue, disregarding possible structural rearrangements in the backbone. As an example, I-Mutant 2.0 [14] represents variants as a vector with 42 dimensions: two for pH and temperature, 20 for encoding the wild-type and mutant residues, and 20 to describe the residue frequency in the environment surrounding the amino acid. Similarly, two versions of MuPro [16] use vectors with 140 elements to encode the residue in a sliding window that considers 3 positions on the left and right of the mutant amino acid. Both methods trained a Support Vector Machine for classification and regression purposes with the radial basis function kernel [18]. This is a general trend of ML-based approaches for stability prediction: non-linear functions are preferred due to their increased ability to detect patterns in the data, leading to better performance. In addition, all methods try to encode explicitly information about the protein of interest using either structure or sequence information. Both information can be described effectively using residue-residue interaction networks (RINs), as suggested by RING [19]. RINs are a graph description of protein structures where nodes represent amino acids and edges represent different types of physico-chemical bonds (e.g. hydrogen bonds, salt bridges, hydrophobic contacts). Using RINs can be of interest for stability estimation due to their implicit detailed representation of different chemical interactions in proteins. These interactions play a central role for the internal folding energy, so they may introduce new discriminative variables for the analysis of mutants [20]. Using this insight, in our work we trained a non-linear neural network for the prediction of stability changes based on RINs. We will show that using this effective protein representation there is an improvement in the prediction of protein stability. We believe that NeEMO can contribute significantly for the characterization of un-annotated missense variants and for protein mutagenesis studies, increasing the knowledge in this challenging field.

## Methods

### Dataset

For machine learning methods, the construction of a dataset is a critical process requiring a meticulous selection and curation of the starting data. The ProTherm database [7] represents a reference dataset describing the effects of amino acid mutations in terms of thermodynamic energy changes, currently containing information on 647 different proteins. Roughly one third of the 22,713 entries represent the Gibbs free energy of the wild type protein, while the reminder report the ΔG of a mutant. It is clear that there is a remarkable redundancy of information that needs to be managed. Here, we decided to focus on the curated version of ProTherm used to train PoPMuSiC 2.0 [17]. In order to avoid bias, we evaluated sequence similarity on the 131 proteins of this training dataset. Using PANADA [21], clustering at 90% and 40% identical sequences produces 129 and 119 different clusters respectively. In particular, none of these clusters had more than three sequences in it. This high diversity is therefore a key factor for the machine learning procedure, as it is likely to provide an effective estimation of the data model.

**Figure 1:**
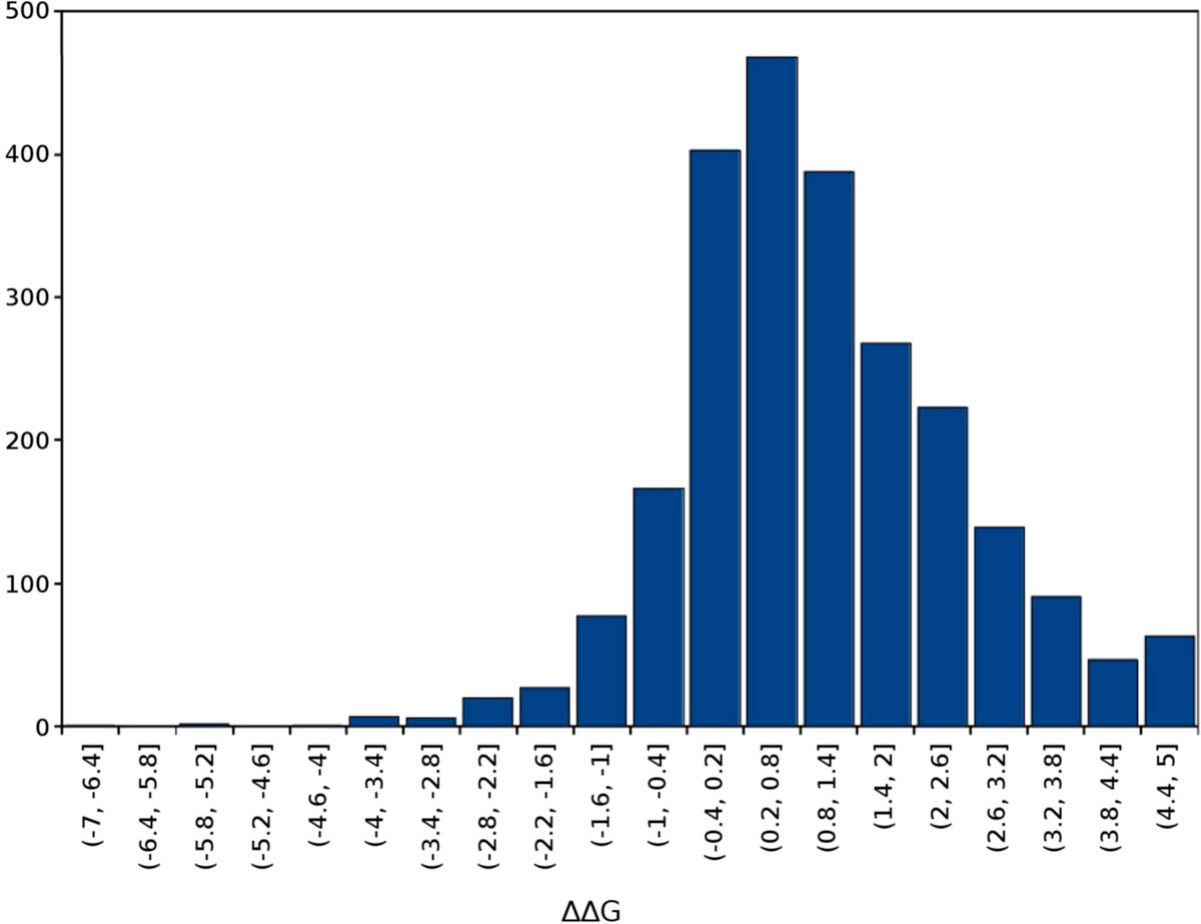
**03940394G distribution on the training set**.

This dataset is particularly informative because it corrects misinterpretations of the original papers and considers only single-site protein variants with known structures that are meaningful for mutation prediction. It should be noted that none of the variants involves either prolines or mutations that destabilise the structure by more than 5 kcal/mol, as these variants tend to alter protein folding significantly. Due to limitations of RING [19] for the management of PDB files with multiple chains, we focused on 113 proteins and 2,399 mutations. Figure 1 shows the training set ΔΔG distribution, highlighting how destabilizing variants are the most frequent ones and proving that the filtering procedure preserves the correct data distribution.

To perform additional tests, we created a second dataset (IM_631) from the training data used in MuPro [16] and I-Mutant [15], containing 631 new mutations in 30 different proteins, to be used as independent samples providing indication of overfitting. The dataset distribution is quite different from the PoPMuSiC data (Supplementary Figure S1), especially in the frequency of highly destabilizing variants (ΔΔG > 5 kcal/mol). The latter dataset explicitly removed strong mutants likely to yield significant changes to the protein structure, which may represent a threat during the learning process. On the other hand, the IM_631 dataset collects real variants with no prior filtering, so these mutations can be used to evaluate NeEMO without bias. Last but not least, the S350 dataset contains further mutations which are typically used to compare the performances of different methods [17]. This data will be considered to obtain a fair comparison of NeEMO performance with other stability prediction tools.

### Relevant features

Our objective is to investigate how useful RINs are in the context of stability prediction. RINs are potentially interesting because they can be used to detect informative amino-acids in a target protein using standard graph algorithms like Dijkstra's shortest path or PageRank [22]. These networks have been generated by RING [19] with default parameters, i.e. closest atom networks where interactions are reported for residues that have atoms at less than 5 Å. There are four main features that we obtain with this tool, which will be briefly described in the following. For a more detailed description of the features see Supplementary Table S1.

#### Evolutionary information

The overall idea is that evolutionary information can discriminate key residues in the protein, either for stability or functional reasons. NeEMO considers conservation, Mutual Information and its correction Average Cluster Purity as a feature for stability prediction. These values are generated by RING, which generates a multiple sequence alignment using PSI-BLAST [23] on the UniRef90 sequence database and computes several measures reflecting evolutionary information of each residue.

#### Residue conformational propensities

The impact of variants strongly depends on the local environment of each residue in the structure. Classical tools for the evaluation of protein structures can highlight residues with high structural constraints that should not be mutated. In the current implementation, RING uses TAP [24], FRST [25], and QMEAN [26] to estimate the amino acid energy contribution. In particular, these tools evaluate statistical potentials such as all atom distance-dependent pairwise, torsion angle, and solvation potentials. All these numerical terms are included in NeEMO for an accurate description of the mutant context.

#### Amino acid information

The wild type, the mutant and its two adjacent residues in the sequence (left and right) are used to describe protein changes. One-hot encoding is used to represent the sequence information, as it was previously shown to be effective [27]. I.e. the 20 standard residues $r_{i},i\in \left\{1,\ldots ,20\right\}$ are translated into a 20-dimensional vector where the *i*-th element is 1, and the others are 0. In addition, secondary structure and relative solvent accessibility (RSA) defined by DSSP [28] are used to describe the local context.

#### Network topology

Using RING it is possible to distinguish between H-bond, inter-atomic contacts, π-cation, π-π stacks, salt bridges and the atoms involved in these interactions [19]. The standard node parameters described in NetworkAnalyzer [29] are computed on that information and used to describe the mutant and its sequence neighbor (left and right) for stability prediction. Centralities are computed by considering multiple sub-network that consider a single chemical bond at a time. In addition, the network size and frequency of each amino acid type in contact with the mutation position in the RIN were also counted. Neighboring residues are defined as those which have any atom at ≤ 5 Å to any of the atoms from the other residue. The overall idea is to comprehensively assess the network connections, and measure if the mutant is central in the protein graph topology. This information was critically discriminative in previous work [15], [20], so we expect it to be also effective in the context of stability prediction.

Last but not least, pH and temperature are considered during the prediction. All information is stored in 184 dimensional vectors for each mutation. Almost half of the features are needed to describe *amino acid information*, due to the one-hot encoding sparsity with 20 descriptors for every residue.

### Training

**Figure 2:**
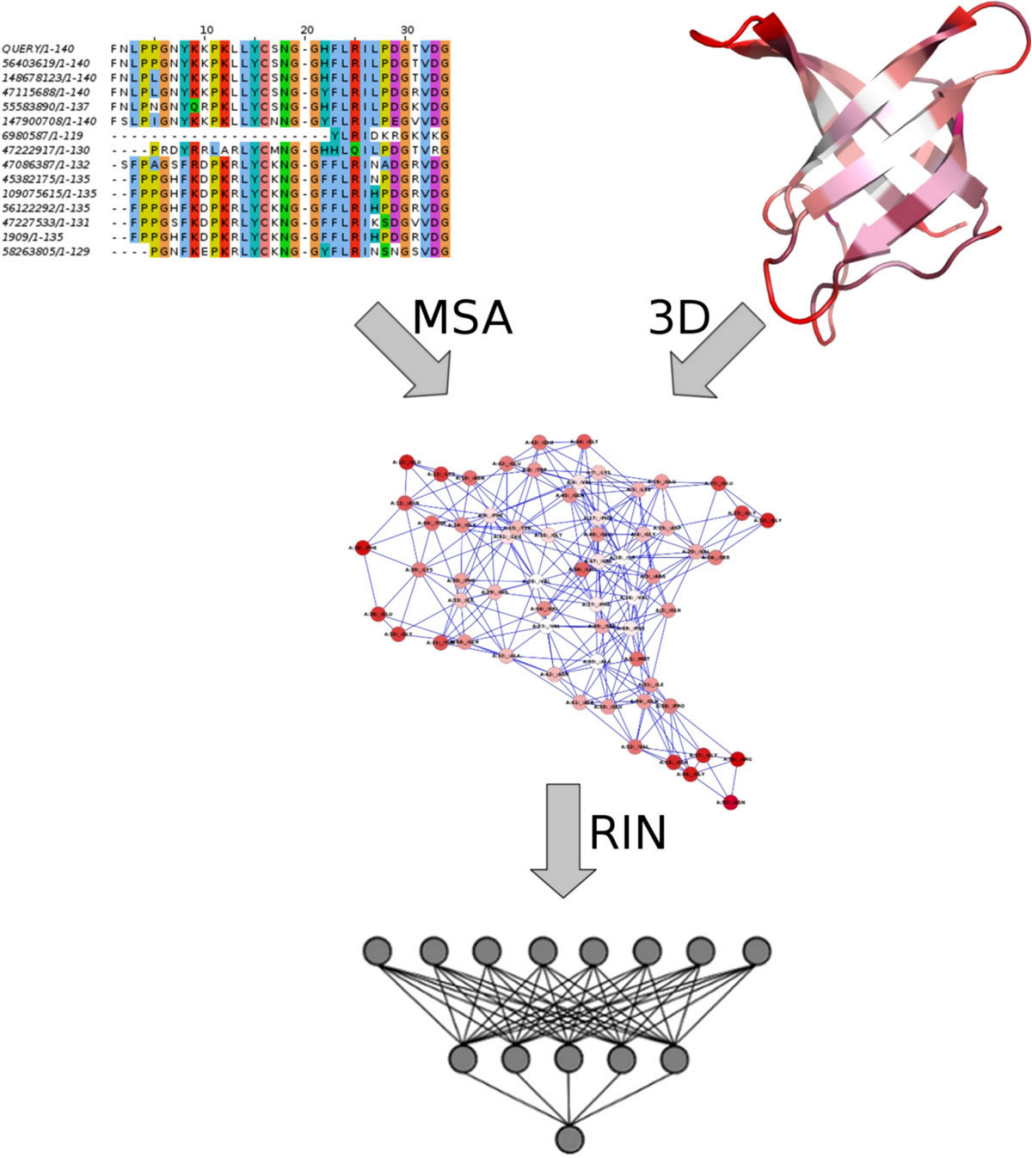
**Representation of the NeEMO pipeline**. The 3D structure of a protein is transformed into a RIN. Node centralities are then computed and combined with other RIN features, such as secondary structure, relative solvent accessibility and sequence conservation. All numerical descriptors are then fed into a neural network that predicts the ΔΔG for the chosen mutation.

Using the encoding described in the previous section, the 2,399 examples were transformed in vectors for training a three-level neural network, with the goal to predict variant ΔΔG values. As shown in Figure 2, the input layer uses RIN information, a single hidden layer is used for non-linear projection of the input data, and a third level is used to estimate the mutation effect in terms of thermodynamic energy. After initial assessment, 5 hidden layer neurons were found sufficient to encode the model data, meaning that the neural network was able to detect a limited number of patterns during the training process which can effectively explain the mutations impact on stability. We used 10 fold cross-validation as implemented in WEKA [30] to estimate the method parameters, i.e. the dataset was randomly split into 10 parts, where 9 were used to train the model and the tenth used as test set. To increase the robustness of the method, 15% of the training data were used as a validation set. During model optimization, the training is stopped once the performance on the validation set does not improve for five iterations. All starting features have non-zero coefficients, so we expect them to be relevant for the final prediction. Three different neural networks we trained. NeEMO uses all 184 features. NeEMO_NOCC does not use network topology and centrality information. Finally, NeEMO_NORING uses only amino acid information, pH, temperature, conservation, QMEAN potential and protein length.

### Performance measures

Several regression and classification measures are computed for a detailed comparison of NeEMO with other methods. Real value ΔΔG predictions are evaluated using standard Pearson correlation *r*:

$$r=\frac{\sum \left(x_{i}-\bar{x}\right)\left(y_{i}-\bar{y}\right)}{\sqrt{\sum {\left(x_{i}-\bar{x}\right)}^{\text{2}}\sum {\left(y_{i}-\bar{y}\right)}^{\text{2}}}}$$

where *x *and *y *represent the real and the predicted energy value. The correlation of the actual ΔΔG ranking is measured against a ranking order induced by the predictions using Kendall's tau (*τ*) and Spearman rank (*ρ*), reporting how predictors differentiate smaller stability changes from bigger ones. Both statistics are calculated as follow:

$$\rho =\frac{\sum \left(r\left(x_{i}\right)-r\left(\bar{x}\right)\right)\left(r\left(y_{i}\right)-r\left(\bar{y}\right)\right)}{\sqrt{\sum {\left(r\left(x_{i}\right)-r\left(\bar{x}\right)\right)}^{\text{2}}\sum {\left(r\left(y_{i}\right)-r\left(\bar{y}\right)\right)}^{\text{2}}}}\;\tau =\frac{CP-DP}{\text{0>.5}\cdot n\cdot \left(n-\text{1}\right)}$$

where ρ is identical to Pearson correlation applied to the rank of the predictions, while τ accounts for the number of prediction pairs having correct order (CP) or wrong order (DP) with respect to the real ΔΔG for the *n *dataset examples. Finally, the standard error σ is used to report the expected distance of the prediction from the real G of the mutation.

### Termophile case study

As an additional test, we consider ten pairs of mesophilic proteins with their termophilic counterparts presented in [31]. In order to compare the stability changes with NeEMO, each sequence pair was first aligned using the Needleman-Wunsch algorithm from the EMBOSS package [32]. The NeEMO energy was then calculated for each aligned residue pair from the mesophilic to thermophilic mutation (MtoT) and vice versa (TtoM). Table 1 lists the 10 pairs of protein structures, their similarity and the pH and temperature at which the ΔΔG was predicted.

**Table 1:** Summary of the 10 pairs of mesophilic and thermophilic proteins used in the case study, their similarity and the environmental conditions (pH and Temperature) used to perform the test [31].

	Mesophile				Extremophile				Alignment	
**Protein**	PDB code	Species	pH	T (°C)	PDB code	Species	pH	T (°C)	Identity	Gaps

Adenylate kinase	1AK2A	*B. taurus*	7	38	1ZIPA	*G. stearothermophilus*	7	65	90/223 (40.4%)	9
Phosphoglycerate Kinase	3PGKA	*S. cerevisiae*	6,6	30	1PHPA	*G. stearothermophilus*	7	65	210/420 (50.0%)	31
Reductase	1LVLA	*P. putida*	7	30	1EBDA	*G. stearothermophilus*	7	65	192/466 (41.2%)	19
Lactate Dehydrogenase	1LDMA	*S. acanthias*	7,9	11	1LDNA	*G. stearothermophilus*	7	65	111/335 (33.1%)	25
TATA box binding protein	1VOKA	*A. thaliana*	7	20	1PCZA	*P. woesei*	7	98	75/198 (37.9%)	22
Subtilisin	1ST3A	*B. lentus*	7	20	1THMA	*T. vulgaris*	6	60	132/282 (46.8%)	16
Carboxy Peptidase	2CTCA	*B. taurus*	7	38	1OBRA	*T. vulgaris*	6	60	93/346 (26.9%)	62
Glyceraldehyde-3-phosphate	1GADO	*E. coli*	7	37	1GD1O	*G. stearothermophilus*	7	65	194/335 (57.9%)	6
Neutral Protease	1NPCA	*B. cereus *	7	30	1THLA	*B. thermoproteolyticus*	7	80	231/318 (72.6%)	2
Phosphofructo Kinase	2PFKD	*E. coli*	7	37	3PFKA	*G. stearothermophilus*	7	65	172/320 (53.8%)	20

## Results

We developed NeEMO, a machine learning method that uses RIN information, to evaluate the impact of amino acid changes in protein stability. Using a curated ProTherm dataset, 10-fold cross validation was used for training and performance evaluation. Finally, the tool is tested on two independent sets of protein variants, providing an unbiased evaluation of its reliability and a fair comparison with other methods.

### Training and cross-validation

NeEMO was trained on a large dataset previously used by PoPMuSiC 2.0 [17]. The results of 10-fold cross validation on this dataset are shown together with a preliminary comparison to other methods in Table 2. Our goal was to assess if the features and the mathematical model of our method are able to fit effectively into the traning data. Several state-of-the-art methods were used, namely Auto-Mute, I-Mutant 2.0 and 3.0, MuPro and PoPMuSiC 2.0. The comparison was not straightforward, as most predictors were occasionally not able to make a prediction for some variants due to their inability to manage certain PDB files. We decided to compare NeEMO only on the mutations where all tools were executed successfully. In many cases the variants of this test set are part of the training dataset of other methods. For this reason, this performance comparison cannot be considered unbiased, and therefore it is just a mean to measure if the fitting procedure is as good as the one used on other methods. As shown in Table 2, NeEMO performs consistently well compared to other state-of-the-art tools. Auto-Mute is the only method providing comparable results, but seems very poor in the input and mutation management, as the method cannot make a reliable prediction for half of the examples (e.g. NMR solved proteins, or in case if atoms with repeated coordinate sets). In view of the good performance in the cross-validation, we expect that the fitting process was overall good.

Interestingly, it seems that NeEMO performs particularly well for amino-acids on β strands (Supplementary Table S3). This improvement is of particular interest, as it suggests that our method can capture and model accurately long range interactions that typically occur in these secondary structures. In addition, performance on buried residues and on coils (see Supplementary Tables S4 and S6) indicate that the method performs very well compared to other methods, confirming that network topology contributes significantly to a proper description of the local amino acid context. On the other hand, NeEMO performance for α helices and exposed residues (Supplementary Tables S2 and S5) are comparable to other methods. As a results, we believe that the training process was successful, suggesting that chosen features and neural networks are a good model of the data.

**Table 2:** Regression performance comparison of NeEMO with other methods on the ten-fold cross-validation test.

		*r*		*ρ*		*τ*	
**Method**	**Mutations**	**Method**	**NeEMO**	**Method**	**NeEMO**	**Method**	**NeEMO**

Auto-Mute	1,144	**0.691**	0.640	**0.686**	0.635	**0.509**	0.456
I-Mutant 2.0	2,171	0.642	**0.678**	0.623	**0.652**	0.467	**0.471**
I-Mutant 3.0	2,112	0.620	**0.679**	0.623	**0.658**	0.434	**0.477**
MuPro	2,398	0.606	**0.665**	0.571	**0.643**	0.416	**0.465**
PoPMuSiC 2.0	2,399	0.623	**0.666**	0.617	**0.644**	0.445	**0.465**

The evaluation is performed only on mutations where both methods were able to make a prediction. In addition, the other methods are likely to have used the test samples in their training.

### NeEMO in-depth analysis

**Figure 3:**
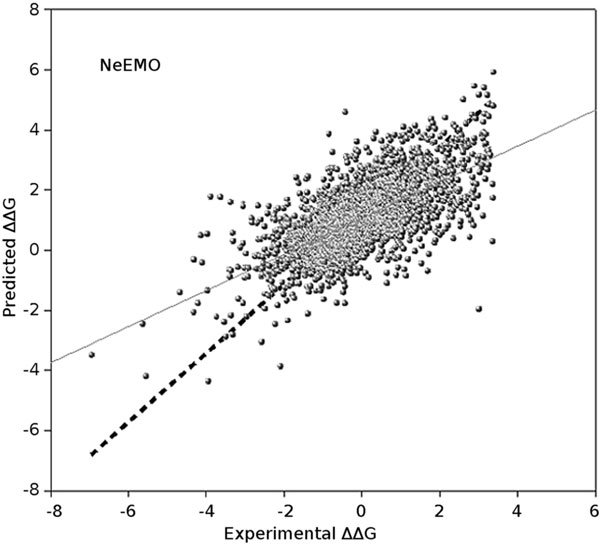
**Regression results of NeEMO versions on the training set**.

In order to test the contribution of the 184 mutation descriptors, we compare the performance of NeEMO, NeEMO_NOCC and NeEMO_NORING on the IM_631 dataset. As can be seen in Figure 3, NeEMO regression has a steep slope that confirms the effectiveness of the training. NeEMO_NOCC and NeEMO_NORING decrease performance (Supplementary Figure S2), showing larger errors for mutations producing a higher stability increase. As expected, the quality of the ΔΔG estimation decreases when less information is provided, suggesting the need of RIN data for good predictions. To study how performance varies for mutations in different conditions, we divided the cross-validation test set into subsets containing only mutations in each of the three secondary structure states (α, β, coil) and computed the class-specific performance (see Table 3). While mutations on α helices show a similar performance compared to the entire dataset, larger differences are found for mutations in β-strands and coils. The correlation for mutations in β-strands is much higher than for any other subset, while performance on mutations occurring in coils shows the lowest results. This was expected, because coil residues tend to be on the surface of globular proteins. Having a tendency towards mobility, they are believed to be regions where unfolding begins. Increased coil mobility facilitates solvent exposure, leading to a reduced number of interactions and hence a lower contribution in RINs. Table 3 shows a similar result for solvent exposed (E, RSA > 25%) and buried (B, RSA ≤ 25%) mutations. In this case, despite NeEMO working better on buried mutations, the difference is less marked. This suggests that secondary structure context is probably the most important feature for stability prediction upon mutations. Last but not least, use of RING network information significantly improves prediction quality in all experiments. On the IM_631 dataset the *r, ρ *and *τ *correlations are 0.63, 0.60 and 0.43 respectively. Considering how the dataset contains unseen mutations, a small drop in performance is expected. It shows that there is no overfitting, and that our network features describe the effect on stability of single amino acid variants. We expect that NeEMO can also perform well in other datasets with very different proteins.

**Table 3:** Correlation measure performance of different NeEMO versions on the IM_631 dataset.

		r	ρ	τ
All	NeEMO	0.666	0.644	0.465
	NeEMO_NOCC	0.637	0.626	0.447
	NeEMO_NORING	0.618	0.603	0.430

Helix	NeEMO	0.645	0.612	0.436
	NeEMO_NOCC	0.613	0.607	0.430
	NeEMO_NORING	0.585	0.600	0.424

Beta	NeEMO	0.716	0.687	0.506
strand	NeEMO_NOCC	0.694	0.672	0.490
	NeEMO_NORING	0.690	0.662	0.482

Coil	NeEMO	0.581	0.588	0.418
	NeEMO_NOCC	0.546	0.560	0.391
	NeEMO_NORING	0.502	0.501	0.350

Exposed	NeEMO	0.603	0.551	0.391
	NeEMO_NOCC	0.553	0.516	0.360
	NeEMO_NORING	0.522	0.498	0.350

Buried	NeEMO	0.638	0.614	0.441
	NeEMO_NOCC	0.612	0.593	0.422
	NeEMO_NORING	0.591	0.559	0.397

NeEMO uses all input features, NeEMO_NOCC does not use node centralities, NeEMO_NORING does not use any RIN feature. Comparisons are shown for the entire dataset, on each of the 3 different secondary structure states and occurring in amino acids exposed to the solvent (e, RSA > 25%) or buried (b, RSA <= 25%).

### Comparison with other methods

We compare the performance of NeEMO with several state-of-the-art methods, namely MuPro [16], two versions of I-Mutant [14][15], PoPMuSiC 1.0 and 2.0 [33][17], Automute [13], Eris [34], CUPSAT [35] and Dmutant [36]. In order to provide an unbiased evaluation of effectiveness, the S350 dataset [17] was used, as it contains mutations unseen to each method. NeEMO was re-trained in order to exclude examples that overlap with the training set. Performance in Table 4, are reported (a) for all the mutations that a single tool can evaluate, (b) for the maximal set of 299 mutations where all predictors are able to make a prediction and (c) for the maximal dataset where we additionally remove 10% of the outliers (leading to 264 mutations). In the latter dataset, outliers are selected automatically for each method as those having the largest residuals in the regression of predicted-observed G values. As can be seen, performance clearly suggests that NeEMO is able to outperform most methods, proving the validity of the training strategy and the strong impact of using residue-residue interaction network data as a tool to study the mutation impact on protein stability. PopMusic2.0 is the only tool with comparable performance, but the unbiased cross validation correlation shown in Table 2 suggests that NeEMO is considerably better on a larger set. The comparison is suitable as both methods trained on exactly the same dataset, so it should give a fair comparison of the predictors. It is also interesting to note that the NeEMO performance is basically the same in both the S350 and cross validation sets, while PoPMusic2.0 has a drop in performance. NeEMO is overall reliable and shows a very good performance on different structure types, like β strands or buried residues (data not shown). RINs seem a clear contribution for the ΔΔG prediction of variants, and NeEMO can be useful for variant annotation.

**Table 4:** Performance of different methods on the independent S350 dataset.

	All mutations			Common mutations			Common mutations -10%		
	
	n	*r*	σ	n	*r*	σ	n	*r*	σ
Automute	315	0.46	1.42	299	0.44	1.45	264	0.60	1.06
CUPSAT	346	0.37	1.46	299	0.37	1.50	264	0.50	1.10
Dmutant	350	0.48	1.38	299	0.46	1.44	264	0.63	1.05
Eris	334	0.35	1.49	299	0.35	1.52	264	0.55	1.07
I-Mutant 2.0	346	0.29	1.50	299	0.27	1.56	264	0.39	1.16
I-Mutant 3.0	338	0.53	1.35	299	0.53	1.37	264	0.71	1.00
MuPro	350	0.41	1.43	299	0.41	1.48	264	0.49	1.12
PoPMuSiC 1.0	350	0.62	1.23	299	0.63	1.26	264	0.72	0.93
PoPMuSiC 2.0	350	0.67	1.16	299	0.67	1.21	264	**0.80**	**0.86**

NeEMO	350	**0.67**	**1.16**	299	**0.68**	**1.19**	264	0.79	0.88

The comparison is reported (a) for all the mutations in the dataset, (b) the maximal subset of mutations where each tool is able to make a prediction and (c) the maximal subset where 10% of outliers are removed. The number of mutations (n) is shown together with the Pearson correlation (*r*) and distance from the real *ΔΔ*G values (σ). The best prediction in each column is shown in bold.

### Termophile analysis

**Figure 4:**
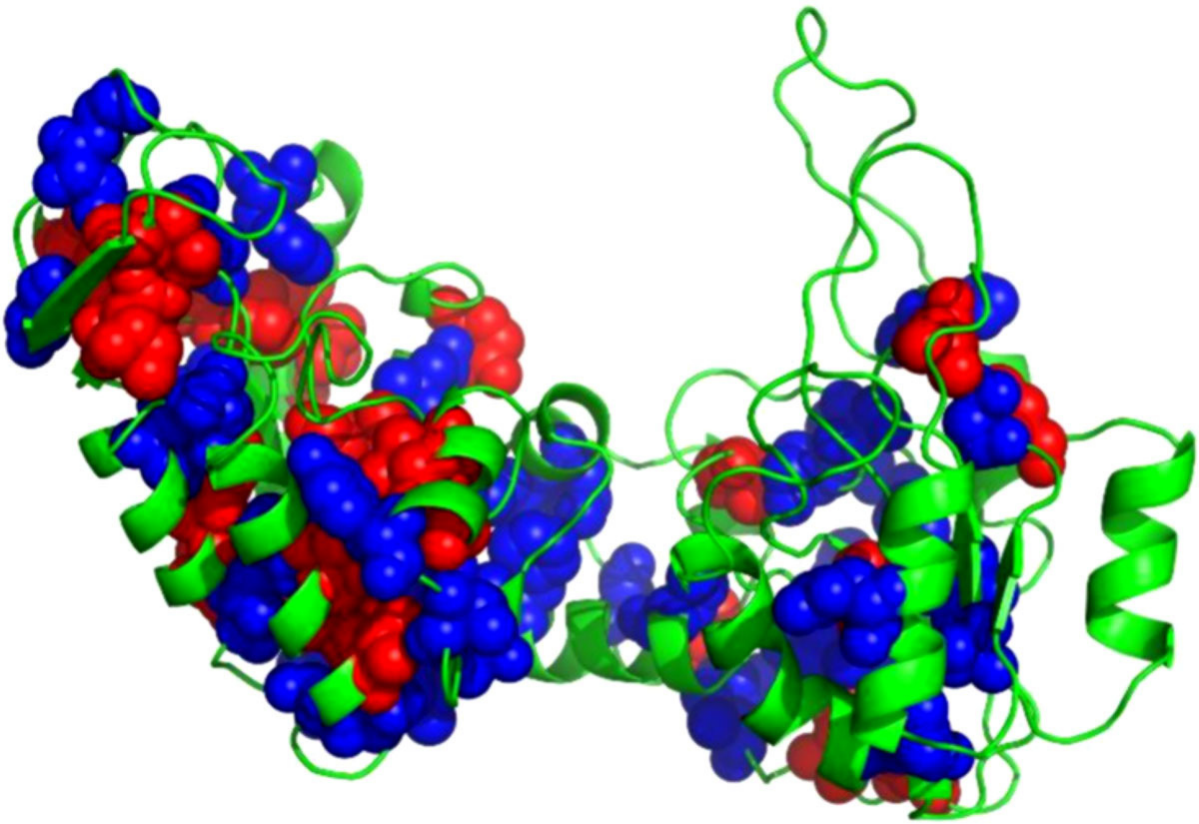
**3D structure of the 3PGKA, showing well predicted buried residues (blue) and mispredictions (red)**. For this protein, the mesophile and thermophile core amino acids share a similar structure.

Effective stability predictors can be used to investigate aspects of biology ranging from protein design to organism evolution. As a proof of principle for NeEMO, we analyzed ten proteins from mesophilic organisms and the correspondent homologs in thermophilic organisms [31]. The simple hypothesis to test is that variants found in termophilic proteins increase stability, while mesophilic variants have the opposite effect. Performing these experiments is complicated by the presence of insertions and deletions in the amino acid sequences which cannot be easily interpreted. NeEMO was used to predict the stability changes upon termophile to mesophile (T**→**M) and mesophile to termophile (M**→**T) for each alignable residue. As shown in Table 5, the results are encouraging. In 66% of T**→**M variants our simple hypothesis seems confirmed (53% of exposed and 76% of buried positions), leading to an expected stability decrease. Overall, the sum of predicted 22062206G also confirms the mutant tendency to reduce stability. In the M**→**T dataset, the expected change in folding energy is not as marked, but there is still an interesting signal. For 6 of the 10 proteins there is a majority of variants predicted to increase stability. This is also confirmed in the sum of predicted 22062206G, where 56% of the mutations support the hypothesis of increased stability. Surprisingly, 68% of exposed positions seem to reduce protein stability, while just 44% of buried residues increase stability. This is in contrast with the T**→**M dataset, and could be due to the highly divergent structures of some proteins. The well predicted Phosphoglycerate Kinase (Figure 4) shows little divergence in the two PDBs. In contrast, protein pairs with unclear support for our hypothesis tend to have divergent 3D structures. Overall, NeEMO seems to be useful in this proof of principle, evaluating a simple hypothesis on stability change in termophiles. Although a more thorough investigation will be necessary to confirm the generality of these observations, it nevertheless provides evidence that NeEMO can be used to prioritize mutagenesis experiments and may be used to support protein design studies.

**Table 5:** NeEMO predictions on the mesophilic and thermophilic mutations.

			T → M			M → T	
		
Mesophile	Thermophile	Increase	Decrease	Energy	Increase	Decrease	Energy
1AK2A	1ZIPA	28	**85**	56.66	50	63	38.92
3PGKA	1PHPA	66	**104**	55.22	**128**	42	-42.47
1LVLA	1EBDA	102	**138**	50.85	**169**	71	-63.93
1LDMA	1LDNA	46	**140**	94.32	**105**	81	-1.99
1VOKA	1PCZA	18	**77**	78.02	42	53	11.63
1ST3A	1THMA	35	**90**	75.91	48	77	34.62
2CTCA	1OBRA	73	**108**	51.41	**100**	81	9.46
1GADO	1GD1O	50	**82**	23.93	**78**	54	-14.69
1NPCA	1THLA	20	**61**	33.95	**49**	32	-2.37
2PFKD	3PFKA	60	**69**	20.48	50	79	41.91

	**Total**	498	**954**		**819**	633	

Amount of reciprocal variants in mesophilc and thermophilic predicted to increase or decrease the stability of the 10 proteins, and their cumulative energy. Cases where predictions support our hypothesis of symmetric ΔΔG behavior of variants are highlighted in bold.

### Web server

The NeEMO web server is freely available to the scientific community from URL: http://protein.bio.unipd.it/neemo/. Once a PDB file is specified by the user, the service computes the RIN in a few minutes, and provides a user-friendly interface for variant prediction. Multiple amino acid changes can be tested at a time, including different pH and temperature parameters. The tool is also very fast. Once the multiple alignment is computed, the effect of a residue change on the protein structure can be predicted in few seconds, making it scalable for large-scale usage.

## Conclusions

NeEMO represents a novel approach to predict ΔΔG changes after point mutations in protein structures. It takes advantage of RINs created by our previous work RING [19] to describe protein structures and interactions between the amino acids forming them. In RINs each residue is described by several features, including secondary structure, solvent accessibility, conservation and a number of residue-specific energy potentials. RING also provides detailed information about interactions found between different amino acids, including their occurrence and types. The interactions present in the RIN are used to compute node centralities that encode the relevance of each RIN node in a protein structure. Inclusion of RINs and information derived from them was shown to improve mutation stability prediction performance. Overall, NeEMO seems able to significantly outperform all other tested methods, and shows very good accuracy across different secondary structures and in classification. It also seems good in terms of reliability, as it can manage and produce a prediction for nearly all PDB files of the PoPMuSiC 2.0 dataset. For the near future, we are planning to extend NeEMO to map multiple chains directly into an integrated RIN.

Another advantage of our approach is that it does not rely on 3D models for the mutant proteins. Instead the RIN for the wild type protein is used to predict the stability change. Other methods have to model the mutant structure first, which may be computationally expensive and in some cases can introduce errors that our protocol avoids. In addition, RINs are very comprehensive data structures that help the management of heterogeneous information sources like evolutionary and topological data. In fact, experiments show that network data improves prediction quality without exception. Finally, it is interesting to note that the evaluation on unseen examples in IM_631 results in basically unchanged performance. This is a nice result, because the ΔΔG distribution of the training data was significantly different. The overall results also prove no overfitting was introduced in NeEMO, and confirm that it can be used effectively for the assessment of mutation impact. As the number of known variants and PDB structures in different organisms is rapidly increasing, we believe that the tool can be important for variant assessment. Finally, NeEMO can also play a role for pathogenicity prediction as shown in [37]. It is well known that stability loss in proteins like *TP53 *[6] is associated with disease development. The ability of RINs to describe proteins and their variants effectively can play a role for the detection of deleterious protein changes, and may also contribute to pathogenicity prediction.

## Authors' contributions

MG, AJMM and SCET designed the study. MG and AJMM performed the experiments. MG, AJMM and SCET analyzed the results. IW programmed the web server and contributed to the machine learning part. MG, AJMM, CF and SCET wrote the manuscript. CF and SCET provided scientific guidance for the project. All authors have read and approved the final manuscript.

## Competing interests

The authors declare that they have no competing interests.

## Declarations

The publication costs for this article were funded by a grant from FIRB Futuro in Ricerca [RBFR08ZSXY], AIRC [MFAG 12740] and CARIPLO [2011/0724].

This article has been published as part of *BMC Genomics *Volume 15 Supplement 4, 2014: SNP-SIG 2013: Identification and annotation of genetic variants in the context of structure, function, and disease. The full contents of the supplement are available online at http://www.biomedcentral.com/bmcgenomics/supplements/15/S4
